# Burlap and buddies: the effects of social enrichment (preweaning mixing) and object enrichment (burlap) on piglet performance, behavior, and welfare in the preweaning environment

**DOI:** 10.1093/tas/txae053

**Published:** 2024-04-09

**Authors:** Ashlyn Scott, Arielle Le Heiget, Reyna Stefanson, Jamie Ahloy-Dallaire, Meagan King

**Affiliations:** Department of Animal Science, University of Manitoba, Winnipeg, MB, Canada, R3T 2N2; Department of Animal Science, University of Manitoba, Winnipeg, MB, Canada, R3T 2N2; Department of Animal Science, University of Manitoba, Winnipeg, MB, Canada, R3T 2N2; Department of Animal Science, University of Manitoba, Winnipeg, MB, Canada, R3T 2N2; Département des sciences animales, Université Laval, Québec, QC, Canada, G1V 0A6; Department of Animal Science, University of Manitoba, Winnipeg, MB, Canada, R3T 2N2

**Keywords:** behavior, enrichment, performance, piglet, preweaning, welfare

## Abstract

When weaned in commercial operations, piglets are not only separated from their sow but also mixed with unfamiliar pigs in an unfamiliar environment with a new diet. These abrupt changes can be stressful for piglets, often having negative welfare consequences. Our objective was to study the effects of early-life preweaning socialization and object enrichment in the preweaning environment. We compared piglet performance, behavior, and welfare across six treatments that combined multi-litter group size (1 vs. 2 vs. 4 litters) and burlap (yes vs. no). We recorded piglet behavior and lesion-scored sows and piglets. Normal conforming data, expressed per experimental unit (and behavior data were averaged over time), were analyzed by ANOVA. When given the opportunity in the sow barn, piglets in multi-litter groups socialized with other litters. Burlap use (*P* = 0.08) was observed in < 5% of the observations but tended to increase in mixed litter groups. Cross-sucking was observed in ~12% of the observations within mixed litter groups and tended to increase with mixed group size (*P* = 0.08). While there was no difference in the proportion of piglets nursing (*P *> 0.10), piglets were less active in the single crate groups and most active in the groups of two litters mixed (*P *= 0.03). Additionally, observed piglet/sow biting behaviors did not differ between treatments; however, piglet-piglet biting (*P* = 0.07), and pen object manipulation (*P* = 0.07) tended to be observed more frequently in non-enriched groups. Piglet displacements were observed more often in non-enriched groups around the pen (*P* = 0.03) but tended to be observed more often in enriched groups at the teat (*P* = 0.07). Preweaning socialization and object enrichment had no impact on the average number of piglets weaned per sow or total piglet mortality (*P* > 0.10). However, the proportion of laid-on piglets decreased as the number of mixed litters increased (*P* = 0.02). The average number of lesions per piglet did not differ between treatments. Although the final lesion scores of sow teat and udder condition did not differ between treatments (*P* > 0.10), sow udder scores tended to worsen more in the single litters than in the mixed litters (*P* = 0.08). Overall, social and object enrichment allows piglets to socialize at a younger age and to redirect their attention toward an object (burlap) which does not negatively impact piglet or sow performance, or behavior, and may improve piglet welfare around weaning.

## Teaser

Providing piglets with preweaning social or object enrichment, respectively, allows them to socialize with conspecifics from other litters at a younger age or redirect their aggression toward an object such as burlap which can lead to improved piglet behavior and welfare before weaning. These methods are feasible in commercial facilities, highlighting the applicability of this research in commercial conditions.

## Introduction

In commercial swine operations, piglets are typically housed with their sow and littermates for approximately 21 d from birth until weaning. During their time with their sow, social groups remain relatively stable as piglets do not typically have the opportunity to socialize with unfamiliar pigs until they are weaned. Upon weaning, piglets are separated from their sow, transported to a new environment, fed a new diet, and mixed with unknown pigs. These stressors can affect pig performance, behavior, and welfare throughout the production system (Ko et al., 2020). Therefore, reducing piglet stress around weaning by implementing enrichment (social and object) preweaning has the potential to benefit the entire production system. To effectively reduce the negative impacts of stress at weaning, interventions should be cost-effective and applicable in commercial settings, and they should encourage piglets’ natural behaviors. The two most practical forms of enrichment for piglets are thus: (1) early socialization (social enrichment) through mixing with other litters (Peden et al., 2021), and (2) object enrichment (Godyń et al., 2019).

Shortly after birth, piglets’ first experience forming a social hierarchy is when they establish a teat order by selecting a teat and, for the remainder of their nursing period, defending it as theirs (Chou et al., 2022). Their hierarchy will likely remain stable until they are weaned and regrouped in the nursery, where increased aggression (displacements, biting, and fighting) and associated lesions are common (Pitts et al., 2000; Ko et al., 2020). Providing piglets with the opportunity to socialize with unfamiliar pigs in the preweaning environment may allow them to develop skills that they can use later in life when they are inevitably regrouped. When piglets are socialized preweaning, their subsequent behavior (after weaning) reflects a more relaxed state (Morgan et al., 2014) and they engage in fewer agonistic behaviors (Hessel et al., 2006; Ledergerber et al., 2015; Salazar et al., 2018) compared to pigs mixed for the first time at weaning. Although there is no known relationship between the age when piglets are mixed before weaning and aggression, another potential benefit of early socialization is that the piglets are younger, smaller, and have less potential to inflict serious harm to other pigs if any agonistic behaviors do occur (Pitts et al., 2000; Van Kerschaver et al., 2021). It could also promote the development of social skills which can set the piglets up for a successful weaning transition, thereby improving piglet behavior, and welfare around weaning. Several researchers have evaluated the effects of mixing piglets before weaning, but they did so only once piglets were a minimum of a week old (D’Eath, 2005; Hessel et al., 2006; Camerlink et al., 2018; Salazar et al., 2018) and also only mixed groups of 2 or 3 litters preweaning. Additionally, some studies may have pseudo-replicated samples or confounded results with other treatments (Turpin et al., 2017; Ji et al., 2021). However, a recent review estimated that preweaning socialization is a low-cost, feasible system to implement on farms to reduce pig aggression (Peden et al., 2021). One potential concern with mixing litters would be reduced sow udder/teat condition and increased piglet mortality due to potential disease transmission. There is, therefore, a need for more robust evaluation of the effects of preweaning mixing of piglets less than 1 wk of age in groups larger than 2 or 3 litters while monitoring mortality and sow udder/teat condition.

Given that piglets are naturally curious animals who begin to explore their environment at a young age (Swan et al., 2021), providing object enrichment in the form of a burlap sheet encourages piglets to engage in their natural exploratory, chewing, and rooting behaviors (Ledergerber et al., 2015; Schmitt et al., 2020; Fynn et al., 2021). Burlap sheets meet five of the “Six Ss” for selecting enrichment put forth by the National Farm Animal Care Council of Canada (NFACC, 2021) by being safe, sanitary, soft, simple, and suspended. The burlap sheets were 100% natural, untreated jute plant fibers woven together which is low risk if consumed by piglets. A burlap sheet may be the optimal form of enrichment for piglets because it poses minimal risk to the manure systems and does not require additional biosecurity measures as would straw or reusable rubber toys, respectively. If pigs are provided with an outlet for their natural behaviors, they may redirect any destructive behaviors or chewing/biting on their pen mates to the burlap (Yang et al., 2018). Providing enrichment may encourage play, reduce agonistic behaviors between piglets, and improve piglet welfare postweaning (Ledergerber et al., 2015; Yang et al., 2018; Schmitt et al., 2020; Fynn et al., 2021).

It is currently unknown if object enrichment and preweaning socialization are additive or synergistic in commercial settings as current literature compares groups of piglets that were socialized and provided enrichment to control groups of piglets that were not socialized or provided enrichment (Ko et al., 2020) or evaluate the effects of either mixing or enrichment but not both. Therefore, the objective of this study was to evaluate the effectiveness of preweaning mixing and object enrichment (in the form of burlap) to reduce weaning stress in piglets in commercial operations. We hypothesized that providing piglets with preweaning socialization and/or a burlap sheet, both pre-and postweaning, would increase the agonistic behavior among piglets preweaning, but improve pig performance and welfare after weaning.

## Materials and Methods

This study was conducted during the summer of 2022 (June—August) at a commercial farrow-to-wean facility housing 6,000 sows, located in South-East Manitoba, Canada. Experimental factors (social and object enrichment) did not interfere with routine pig care and management procedures, which were conducted by barn staff.

### Animals, Housing, and Experimental Design

All experimental procedures were approved by the Animal Care Committees of the Research Ethics Board (Protocol Reference Number: F21-022, AC11708) at the University of Manitoba, Fort Garry campus.

Of the total 32 farrowing rooms in the facility, we focused on four farrowing rooms on trial per replicate and ran the experiment three times (each with a different group of pigs); however, one was interrupted by extreme weather and had to be terminated prematurely. Of the 26 crates in each farrowing room, 24 were on trial while two were designated as not on trial (N.O.T.) to accommodate any nurse sows/foster-offs after the start of the trial period. The farrowing pens used in this trial measured 2.06  × 1.63 m with a sow crate in the center measuring 1.98  × 0.61 m. All piglets on trial were out of Fast F1 sows crossed to a DNA Duroc 600 boar.

In this study, we used a 3 × 2 factorial arrangement of treatments to assess the effects of: (1) the number of litters mixed preweaning (1 litter, 2 litters mixed, 4 litters mixed) and (2) burlap provision (enriched vs. non-enriched). Litters (*n* = 288) were randomly assigned to one of the six treatments where the size of each experimental unit (considered to be the group of piglets to which the treatment was applied) differed according to the treatment (*n* = 168 total experimental units): 1 litter, not enriched (1N, *n* = 48 × 1 litter), 1 litter, enriched (1E, *n* = 48 × 1 litter), 2 litters, not enriched (2N, *n* = 24 × 2 litters), 2 litters, enriched (2E, *n* = 24 × 2 litters), 4 litters, not enriched, (4N, *n* = 12 × 4 litters), and 4 litters, enriched (4E, *n* = 12 × 4 litters). The estimated minimum sample size of the number of experimental units needed to detect treatment differences was calculated based on previous studies with sample sizes of 15, 12, 20, and 27 (Pitts et al., 2000; Salazar et al., 2018; Van Kerschaver et al., 2021), respectively. Using their treatment means and standard deviation, or to detect a 10% difference among treatments, the sample size needed was calculated to range from 3 to 9 per treatment for behavior and 1 to 14 per treatment for lesions with 80% power and an alpha of 0.05.

The allocation of treatments was randomized within rooms using a random number generator, balanced for sow parity (average of sow parity was the same across treatments [Table 1]), and each treatment was present in each room.

**Table 1.** Sow parity (mean ± SD) across six treatments^1^ comparing object enrichment (burlap provision) and early-life mixing of litters (social enrichment) within each room (block), replicate, and across all replicates^2^ and rooms^3^ within replicates

	1N	1E	2N	2E	4N	4E	*P* value
*Replicate 1*	*4.1 ± 2.1*	*4.3 ± 2.6*	*3.7 ± 1.7*	*5.3 ± 2.4*	*4.1 ± 2.3*	*4.6 ± 2.4*	*0.43*
Room A	3.8 ± 1.0	4.0 ± 1.4	4.0 ± 0.8	6.0 ± 2.2	3.5 ± 1.3	5.5 ± 2.1	0.17
Room B	3.0 ± 2.0	3.3 ± 1.5	3.8 ± 2.1	5.3 ± 1.0	3.3 ± 1.9	5.8 ± 1.5	0.15
Room C	4.5 ± 3.1	3.3 ± 2.6	3.0 ± 2.7	3.5 ± 3.7	5.8 ± 3.6	2.5 ± 1.3	0.67
Room D	5.3 ± 1.7	6.8 ± 3.2	4.0 ± 0.8	6.5 ± 1.9	3.8 ± 1.7	4.8 ± 3.6	0.38
*Replicate 2*	*5.3 ± 2.2*	*4.4 ± 1.8*	*5.3 ± 2.2*	*5.9 ± 2.2*	*5.2 ± 2.2*	*5.1 ± 2.2*	*0.51*
Room A	4.0 ± 2.8	3.3 ± 1.9	4.0 ± 2.7	6.5 ± 4.0	3.0 ± 0	2.8 ± 0.5	0.32
Room B	5.3 ± 2.2	4.4 ± 1.8	5.3 ± 2.2	4.9 ± 2.2	5.2 ± 2.2	5.1 ± 2.2	0.49
Room C	6.3 ± 1.9	6.0 ± 0.8	5.8 ± 1.0	5.3 ± 1.5	5.5 ± 1.0	6.0 ± 1.8	0.92
Room D	6.3 ± 2.6	5.0 ± 1.2	5.5 ± 1.3	6.0 ± 1.4	7.5 ± 2.7	6.8 ± 2.2	0.57
*Replicate 3*	*3.7 ± 1.9*	*4.1 ± 2.1*	*4.8 ± 2.2*	*4.2 ± 2.3*	*5.1 ± 2.1*	*4.8 ± 2.7*	*0.48*
Room D	2.0 ± 0.8	2.8 ± 1.5	3.8 ± 1.9	2.5 ± 1.0	3.5 ± 1.0	4.3 ± 2.1	0.29
Room E	3.7 ± 1.9	4.1 ± 2.1	4.8 ± 2.2	4.2 ± 2.3	5.1 ± 2.1	4.8 ± 2.7	0.57
Room F	5.3 ± 1.7	4.8 ± 2.9	5.3 ± 1.5	5.0 ± 1.8	6.0 ± 0.8	5.5 ± 1.3	0.94
Room G	4.0 ± 1.6	4.5 ± 1.0	4.0 ± 1.6	5.5 ± 3.4	5.0 ± 3.6	5.8 ± 3.9	0.91
Overall	6.7 ± 2.3	7.1 ± 2.4	7.1 ± 2.2	7.5 ± 2.4	7.6 ± 1.8	7.2 ± 2.3	0.49

^1^Expressed per experimental unit, 1 litter, not enriched (1N), 1 litter, enriched (1E), 2 litters, not enriched (2N), 2 litters, enriched (2E), 4 litters, not enriched (4N), 4 litters, enriched (4E).

^2^All replicates: 1N (*n* = 16), 1E (*n* = 16), 2N (*n* = 16), 2E (*n* = 16), 4N (*n* = 16), 4E (*n* = 16).

^3^Per room: 1N (*n* = 4), 1E (*n *= 4), 2N (*n* = 4), 2E (*n* = 4), 4N (*n* = 4), 4E (*n* = 4).

Piglets were on trial for approximately 3 wk in the farrowing rooms until they were weaned at an average age of 22 d (min. 18 d; max. 27 d). Postweaning results are discussed in a companion manuscript (Scott et al., 2024).

The following procedures will be referred to as ‘piglet processing’: all piglets were administered an iron supplement 1-d post-farrowing and male piglets were castrated by trained barn staff, as per their protocols. If needed, barn staff cross-fostered piglets to ensure that there were enough functional teats relative to the number of piglets and to roughly standardize the number of piglets per litter per room, as per standard practice at the barn, prior to the start of the trial (day 0 to 2 of life).

The trial began on trial day 0, which occurred 1 to 3 d after the entire room had farrowed and within 24 h of processing. At this point, we applied our treatments. The plastic panels that divided the farrowing pens between each sow were removed to allow socialization between litters, but before removing the panels, we marked each sow and her piglets with animal-safe paint, by crate, to allow identification of piglets and their respective sows within each experimental unit after mixing occurred. Removing the dividers freed up an average of 65 cm^2^ and 93 cm^2^ of heat mat space per piglet in the groups of two and four litters mixed, respectively. At the same time, two 0.31  × 0.61 m (1ft × 2 ft) burlap sheets were secured using C-clamps at the back of the farrowing pen for the enriched treatment groups, out of reach of the sow, which was an appropriate length to just reach the ground for ease of access for the piglets.

### Behavioral Observations

Two types of in-person behavior observations were made four times on each observation day (twice in the AM, twice in the PM), occurring on trial days 3, 5, 9, 11, and 13 ± 1 d. Scan sampling focused on behavioral states (sucking, active, resting, and using burlap), whereas continuous sampling focused on event behaviors (Table 2).

**Table 2.** Ethogram of preweaning piglet behaviors assessed to compare the effects of object enrichment (burlap provision) and early-life mixing of litters (social enrichment)

Behavior	Description	Behavior, sampling type	References
*Aggressive*			
Fighting	Physical encounter between at least two pigs including head-to-head fights, biting another pig, as well as pushing or knocking another pig with the head causing one pig to retreat/withdraw or both pigs engaging in aggression. May or may not include vocalizations	Event, Continuous	Ledergerber et al (2015) Ji et al (2021)
Tail/Ear Biting	Pigs engaged in oral manipulation of pen mate’s tail or ear, may or may not result in wounds	Event, Continuous	Hakansson & Bolhuis (2021)
Belly-nosing	Piglet engaged in rhythmic nudging of another piglet’s abdomen (belly) with their nose; at least 3 nudges in a row	Event, Continuous	Widowski et al (2008)
Displacement	Physical contact between pigs resulting in one pig losing control over teat/object or needing to move or being pushed out of the way	Event, Continuous	
*Investigative*			
Socializing	Pigs engaged in actions that did not cause the recipient to react negatively. Ex: nudging/sniffing: snout of piglet is used to gently touch another piglet’s or sow’s body, not including any behavior directed at the sow’s udder or piglet’s abdomen	Event, Continuous	Morgan et al (2014) Yang et al (2018)
Pen objects	Nosing, licking, or chewing any object which is part of the pen (e.g., feeder or bar of sow crate), but excluding the enrichment object. Excluding any behavior toward creep feed	Event, Continuous	Yang et al (2018)
Burlap	Manipulating (M) or investigating (I) the enrichment objects (burlap that was deliberately put into the pen by the researcher) with mouth or snout, resulting in visible movement of the target (M) or sniffing or staring at enrichment within 1 foot of burlap (I)	Event, Continuous	Yang et al (2018)
*General*			
Cross-sucking	Piglet from another litter massages or sucks at another sow’s udder	Event, Continuous	
Nursing	Piglet massages or sucks at the [sow’s] udder	State, Scan	Ledergerber et al (2015)
Active	Locomotion (scampering, running, and walking), Climbing (Piglet uses its feet to elevate itself onto the body of the sow or another piglet), Standing (All four legs supporting the body with no ambulation or touching anything with their nose or mouth)	State, Scan	Yang et al (2018) Morgan et al (2014)
Resting	Lying (Whole length of body on the floor or on other pigs, i.e., not supported by their legs) or sitting (Hind quarters on the floor, front legs supporting body)	State, Scan	Morgan et al (2014) Ledergerber et al (2015)

Scan behavior observations were made by counting the number of piglets performing each of the listed behaviors: sucking, active, resting, and using burlap, at the start of each of the four observation times on each observation day. Because resting was the most predominately observed state behavior, nursing, and active results were analyzed as representative averages scaled to the number of piglets resting, with a constant added to eliminate zeros as a denominator (if no piglets were resting), then using the natural log on the calculated ratio (as described in Codd et al., 2003). The transformation can be defined as:


$$\begin{array}{l} logistic-active=\ln[(\%\;active+0.5)/(\%\;resting+0.5)] \end{array}$$

$$\begin{array}{l} logistic\text{-}nursing\;=\;\ln[(\%\;nursing+0.5)/(\%\;resting+0.5)] \end{array}$$

Scan observations also collected piglet movement data in mixed groups by counting the number of piglets of each color in a crate at the time of observation as an indicator of the piglets traveling across the mixed crates (by referencing the sow’s assigned color).

Continuous behavior observations used one-zero sampling, recording two consecutive 30-s observation periods, repeated four times per observation day two in AM (0900 to 1200 hours), two in PM (1300 to 1600 hours). Four trained observers with a moderate or higher interobserver reliability score for each behavior (Table 3) performed in-person observations. The teams of two observers alternated rooms and crates between observation periods so that each team observed each room twice and each member of the team observed each pen twice per day.

**Table 3.** Interobserver Reliability Scores (IOR) for all four behavior observers when assessing the effects of object enrichment (burlap provision) and early-life mixing of litters (social enrichment)

Continuous behavior observations	Kappa	Scan behavior observations	ICC^1^
Fighting	0.70	Nursing	0.73
Biting	0.66	Active	0.91
Displacement	0.58	Resting	0.88
Socializing	0.63		
Pen Objects	0.54		

^1^ICC3 (Intraclass Correlation Coefficient; Model: Two-way; Type: consistency).

### Piglet Lesion Scoring

Lesions were scored using the system presented by Turpin et al. (2017) which assessed the severity of scratches (red marks and scabs) on the piglets’ ears, tail, and body. Lesions were scored by trained observers in teams of two (intraclass correlation coefficient between teams = 0.83 (*intraclass correlation coefficient3 [model: two-way; type: consistency]*) on trial day 13 ± 1 d, as well as on the day before weaning. The number of lesions present in a group of pigs was counted and then the number of lesions (expressed per pig to standardize across different group sizes) was calculated for each experimental unit by taking the total number of (body lesions + tail lesions + ear lesions)/total number of piglets in that unit.

### Sow Lesion Scoring

Sow lesions were scored on trial day 1 and on the day before weaning by a single trained observer for all three batches. Each sow was assigned an udder score and a teat score; a score of 0 indicated that no lesions were present or, if lesions were present, a score of 1 to 3 (depending on severity) as described by Van Kerschaver et al. (2021). Sow teat descriptions were also noted to indicate if the sow had any inflamed, scabbed, amputated, or split teats. Sow lesion scores assessed sow condition over the course of the lactation period on a per experimental unit basis (averaged across sows within groups of mixed litters).

### Litter Performance

Litter weights and the number of piglets weaned per sow were recorded in the sow barn. Initial litter weights and final litter weights were collected on the day before weaning however, weaning weight data were not available from batches 1 and 3 and will therefore not be included in the analysis due to lack of power.

The causes of preweaning mortality were collected by barn staff, using the farm protocols and recording sheets, on a per-crate basis (the number of piglets weaned per sow, and piglet mortality). These were then combined and expressed on a per experimental unit basis to account for the differences in piglet numbers between single, double, and four litter groups.

### Statistical Analyses

All data were expressed per experimental unit and behavior data were averaged over time. An ANOVA was run on a generalized linear model for all data that met the normality criteria using the Bartlett test and Shapiro–Wilks tests on the residuals of the model. The model included ‘room’ and ‘replicate’ to account for any differences between different batches of pigs. Treatment factors (number of litters mixed and enrichment) were included to assess any interaction effects. However, due to a lack of interaction effects for all outcomes, the main effects were therefore reported independently. Final sow lesion scores were analyzed with the initial lesion scores included in the model as a covariate. Significant differences were declared at *P *≤ 0.05 and tendencies were declared at 0.05 < *P *≤ 0.10. If significant differences or tendencies were found, the Tukey post hoc test was used to run pair-wise comparisons between treatments. Non-normal conforming data were transformed or analyzed using the Kruskal–Wallis test. All statistical analyses were run in Rstudio © (Version 2023.03.0 + 386).

## Results and Discussion

### Piglet Behavior

Piglets in mixed litter groups did travel between pens, showing that they were making use of the additional space and socializing with piglets from other litter. While there was no difference in the proportion of piglets nursing (*P *> 0.10), there was a significant mixing effect in terms of the proportion of active piglets (*P *= 0.03); piglets from the single crate groups were the least active (26%, i.e., 2 active, 8 resting), while groups of two litters mixed were the most active (37%, i.e., 6 active, 15 resting). Groups of four litters mixed were intermediate and did not differ from the other groups (33%, i.e., 10 active, 30 resting). Furthermore, a similar proportion of piglets were observed traveling to crates other than their home crate regardless of whether they were in a group of 2 or 4 litters (*P* > 0.10).

Cross-sucking tended to be observed more frequently in the groups of 4 crates compared to the groups of 2 crates (*P* = 0.08) (Table 4). In no previously published study has there been a comparison of different group sizes on cross-sucking occurrence. However, our results are consistent with other studies that found a low occurrence of cross-sucking overall (D’Eath, 2005; Morgan et al., 2014). Furthermore, the early removal of the pen dividers did not negatively impact the formation of the sow-piglet bond given that we observed minimal cross-sucking. Because nursing bouts are typically synchronized between litters nearby (Widowski et al., 2008; Silerova et al., 2013), we observed that piglets responded well to their sow’s vocalizations and generally scurried back to their own sow for nursing. Because the timing of partition removal has not been shown to influence the incidence of cross-sucking among mixed litters (Van Kerchaver et al., 2021), implementing early-life socialization can be tailored to each barn’s preference without risk of negatively impacting the sow-piglet bond. Our results indicate a tendency for larger groups of mixed litters to experience more cross-sucking, however, the occurrence of this behavior did not result in reduced litter performance in these treatment groups (Table 5). Based on a previous study, cross-sucking may not be a result of our applied treatments as much as it is due to sow behavior; Morgan et al. (2014) describe some sows as being complaisant to cross-sucking piglets, frequently allowing “alien” piglets to nurse while other sows will not allow “alien” piglets to cross-suck. Future work could investigate which piglets were cross-sucking to determine if it is primarily smaller piglets that are “opportunistic feeders”, seeking out teats that are available to them, or if it is primarily larger piglets who are “sow hopping” and bullying other piglets off their sows to get more milk. Depending on who is performing cross-sucking, this may influence management decisions for mixed litters in commercial operations.

**Table 4.** Percentage of observations where piglet behaviors were observed across six treatments^1^ to compare the effects of burlap provision (object enrichment) and early-life mixing of litters (social enrichment)

Behavior	1E	1N	2E	2N	4E	4N	Mix factor *P*-value	Burlap factor *P*-value
Burlap use^2^^,^^3^	0 ± 0	N/A	4.2 ± 6.2	N/A	2.1 ± 3.1	N/A	0.08	N/A
Cross- sucking^4^^,^^5^	N/A	N/A	8.3 ± 0.11	12.7 ± 0.11	14.3 ± 0.10	15.1 ± 0.07	0.08	0.18
Biting (piglet-sow)^3^^,^^6^	0 ± 0	8.3 ± 11.1	5.9 ± 7.7	4.2 ± 6.2	5.7 ± 4.6	6.3 ± 2.5	0.52	0.38
Biting (piglet-piglet)^3^^,^^6^	8.3 ± 12.4	11.1 ± 8.7	12.5 ± 5.2	13.9 ± 5.8	12 ± 6.2	11.9 ± 3.4	0.37	0.07
Displacement (pen)^3^^,^^6^	0 ± 0	5.0 ± 7.4	4.76 ± 2.8	5.6 ± 8.2	4.7 ± 3.1	4.6 ± 2.8	0.15	0.03
Displacement (teat)^3^^,^^6^	8.3 ± 6.2	4.6 ± 6.8	5.3 ± 5.8	4.2 ± 6.2	2.9 ± 3.9	4.0 ± 3.4	0.16	0.07
Fighting (pen)^3^^,^^6^	0 ± 0	2.1 ± 3.1	4.2 ± 5.6	4.2 ± 6.2	4.0 ± 3.1	4.7 ± 2.3	0.23	0.52
Fighting (teat)^3^^,^^6^	0 ± 0	0 ± 0	2.1 ± 3.1	4.2 ± 6.2	2.9 ± 2.7	2.3 ± 2.8	0.51	0.57
Pen object manipulation^5^^,^^6^	14.1 ± 15.1	12.3 ± 12.3	7.9 ± 7.9	13.4 ± 6.9	14.3 ± 6.4	13.8 ± 5.7	0.86	0.07
Socializing (piglet-sow)^5^^,^^6^	11.7 ± 10.6	14.7 ± 12.7	17.3 ± 12.0	16.5 ± 10.5	16.2 ± 9.5	15.0 ± 7.3	0.11	0.26
Socializing (piglet-piglet)^5^^,^^6^	13.7 ± 9.8	14.5 ± 10.8	16.3 ± 6.7	15.0 ± 8.0	16.4 ± 7.0	16.7 ± 6.6	0.45	0.69

^1^Expressed per experimental unit, 1 litter, not enriched (1N), 1 litter, enriched (1E), 2 litters, not enriched (2N), 2 litters, enriched (2E), 4 litters, not enriched (4N), and 4 litters, enriched (4E).

^2^1E (*n* = 34), 2E (*n* = 18), and 4E (*n* = 12).

^3^Expressed as median ± MAD; data did not meet the ANOVA assumptions and a Kruskal–Wallis test was used.

^4^2N (*n* = 17), 2E (*n* = 18), 4N (*n* = 12), and 4E (*n* = 12).

^5^Expressed as mean ± SD; data met the ANOVA assumptions.

^6^1N (*n* = 32), 1E (*n* = 34), 2N (*n* = 17), 2E (*n* = 18), 4N (*n* = 12), and 4E (*n* = 12).

**Table 5.** The average litter performance parameters did not differ by treatment^1^ (*P* > 0.01) when evaluating the effects of burlap provision (object enrichment) and early-life mixing of litters (social enrichment).

Treatment	Birth weight per piglet ± SD, kg	Preweaning mortality on trial^2^ ± SD, %	Number of piglets weaned per sow ± SD
1N	2.0 ± 0.41	8.7 ± 7.7	10.9 ± 1.36
1E	1.9 ± 0.52	6.7 ± 7.1	10.5 ± 1.71
2N	1.9 ± 0.32	6.1 ± 5.7	10.3 ± 1.87
2E	1.9 ± 0.31	9.1 ± 7.0	10.5 ± 1.12
4N	1.8 ± 0.26	10.3 ± 4.8	10.7 ± 0.89
4E	1.9 ± 0.22	11.3 ± 6.0	10.3 ± 1.20

^1^ *Expressed per experimental unit,* 1 litter, not enriched (1N, *n = 32*), 1 litter, enriched (1E, *n = 30*), 2 litters, not enriched (2N, *n = 16*), 2 litters, enriched (2E, *n = 16*), 4 litters, not enriched (4N, *n = 8*), 4 litters, enriched (4E, *n = 8*).

^2^From day 2 ± 1 to 22 ± 4 of life.

Within the enriched groups, the frequency of observed burlap interaction (investigating and/or manipulating) tended to increase with the number of crates mixed (*P *= 0.08; Table 4). Burlap use was observed less than 5% of the time, however, the increased burlap use observed in the larger mixed groups could be attributed to social learning and social facilitation among piglets. Giving the sow access to the burlap could also encourage piglets to engage with it; however, more research would need to be done to address risks to manure systems if the stronger, and therefore, more destructive, sow was provided burlap as enrichment alongside her piglets.

If piglets can identify the burlap in the preweaning environment as an outlet for their natural chewing/biting behaviors, they may be less likely to develop agonistic behaviors such as tail/ear biting. Although our results showed that biting of the sow did not differ between treatments, we found that biting between piglets tended to be observed more often in the groups without access to burlap (*P *= 0.07). This supports the assumption that biting could be redirected from pen mates to the burlap. Not only can having access to burlap redirect the biting behaviors between piglets, but it can also redirect the manipulation of objects around the pen. Pen object manipulation, such as nosing, licking, or chewing on the flooring or bars of the sow’s crate, tended to be observed more often in the non-enriched groups compared to the enriched groups (*P* = 0.07). Though there were no displacements observed at the burlap and overall displacements did not differ between treatment groups (*P* > 0.10), piglet displacement around the pen was observed more often in the non-enriched groups compared to the enriched groups (*P *= 0.03) whereas displacement at the teat tended to be observed more often in the enriched groups compared to the non-enriched groups (*P *= 0.07). Indicating a difference in the observed frequency of displacements depending on the location (around the pen or at the teat), but not overall. We speculate that non-enriched groups were observed displacing their pen mates more frequently around the pen as a means to express frustration or boredom, whereas the enriched groups were able to redirect these behaviors towards the burlap, instead of their pen mates, or did not experience the same level of frustration or boredom since they were provided enrichment material. The tendency for enriched groups to exhibit more nursing-related displacements may indicate that the enriched piglets were more teat-motivated. It is expected that the two types of displacements have different motivations because displacements around the pen do not have a clear “goal” or purpose appearing to motivate the piglet to engage in the action (besides moving around the pen) while in contrast, displacements at the teat are more likely to be part of maintaining/ establishing teat order during nursing bouts.

In previous literature, displacements are commonly categorized as aggressive behavior and are not distinguished from fighting (Fels et al., 2021). However, for our observation, we differentiated between them and neither treatment factor had any impact on fighting at the teat or in the pen (*P *> 0.10). Additionally, belly nosing between piglets was seen in less than 1% of observations in the preweaning environment and was therefore not analyzed statistically. In terms of social behaviors, there was no observed difference between treatments for sow-piglet or piglet-piglet socialization (*P *> 0.10).

### Piglet Lesion Scores

Across treatments, there was no difference between the number of lesions per pig (*P *> 0.10) on the day before weaning. This is consistent with the findings presented by Yang et al. (2018) and Ko et al. (2020) regarding object enrichment and/or social enrichment who did not observe any lesion differences preweaning. Piglets in the preweaning environment are smaller and less capable of inflicting injury/lesions on pen mates which is another benefit of mixing piglets earlier in life than at weaning; once piglets reach weaning age, they are larger in size and therefore stronger. Piglets who are provided with the opportunity to socialize with unfamiliar piglets in their familiar preweaning environment acquire skills while the risk of inflicted injury on another is lower. This may continue to benefit the piglet during other mixing events throughout multi-stage production systems, as indicated by many previously published studies (Kutzer et al., 2009; Ko et al., 2020; Fels et al., 2021; Van Kerschaver et al., 2021). Even though it was a concern that socializing piglets early in life (preweaning), instead of waiting until weaning, may pose a greater risk for piglets developing face lesions (Chou et al., 2022), this was not the case based on our preweaned lesion results. Furthermore, 28% of producers who participated in a sow and piglet lesion risk factor survey, that included producers from 17 countries, Chou et al. (2022) identified piglets reared in barren environments to be at a greater risk for developing lesions based on their experiences. Although enrichment resulting in reduced piglet lesions is not reflected in our study or others (Yang et al., 2018; Ko et al., 2020), 38 of the 75 producers surveyed (51%) had implemented enrichment on their own and 33 of the 75 (44%) of them reported it working to reduce piglet face lesions (Chou et al., 2022).

### Sow Lesion Scores

**Figure 1.**
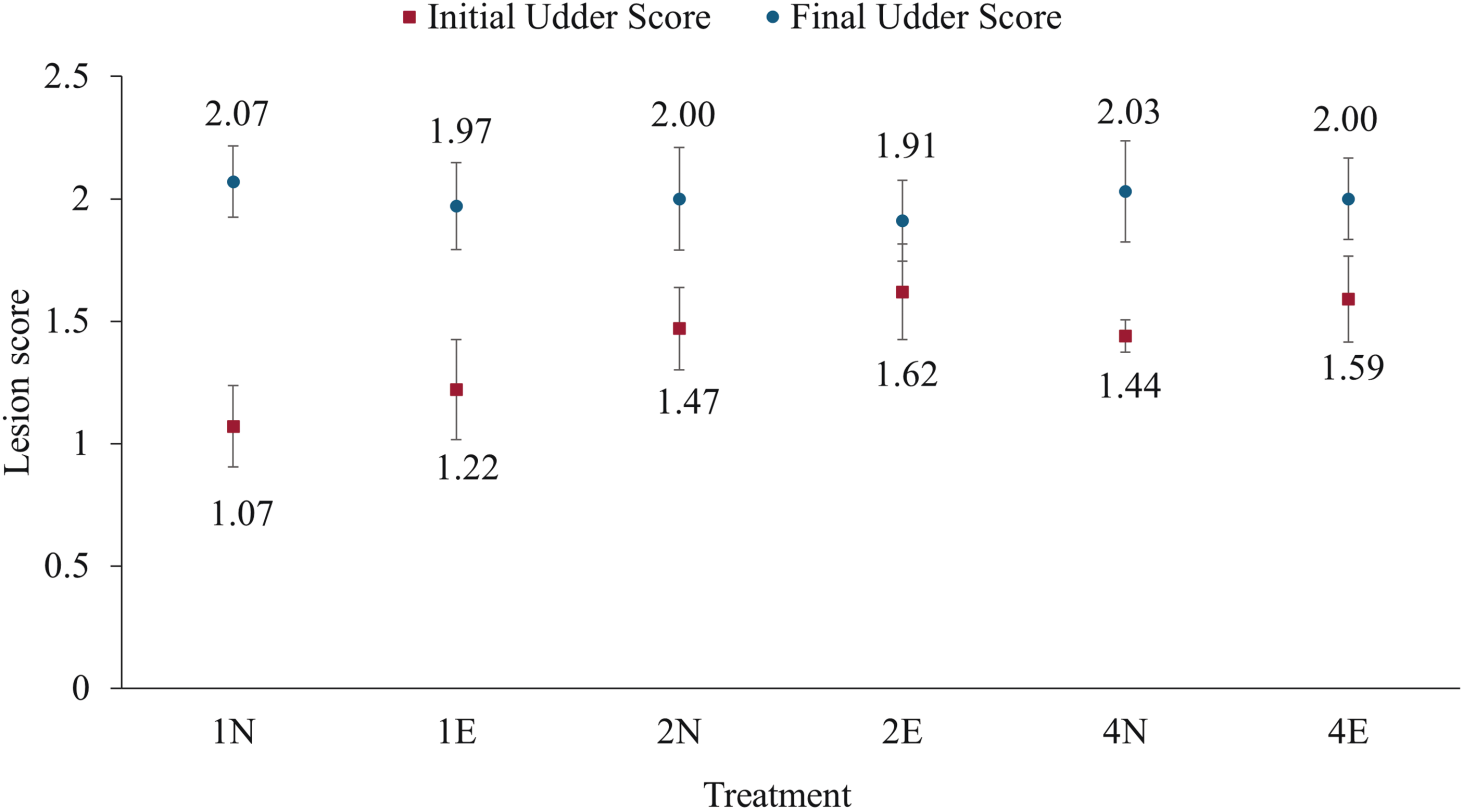
The effects of burlap provision (object enrichment) and early-life mixing of litters (social enrichment) on average initial and final sow udder lesion scores (± SD) by treatment. Initial udder scores scored on day 1 of the trial (*P *= 0.05); final udder scores scored the day before weaning, did not differ by treatment (*P* > 0.10) with initial udder lesion score as a covariate in the model. Scores were based on the following scale: score 0 = no lesions; score 1 = mild lesions; score 2 = moderate lesions; 3 = severe lesions. Sample size of each treatment is as follows: 1N (*n* = 32 × 1 litter, not enriched), 1E (*n* = 29 × 1 litter, enriched), 2N (*n* = 16 × 2 litters, not enriched), 2E (*n* = 16 × 2 litters, enriched), 4N (*n* = 8 × 4 litters, not enriched), and 4E (*n* = 8 × 4 litters, enriched).

While controlling for initial sow lesion scores, final sow lesion scores (scored on the day before weaning) for both udders and teats did not differ between the six treatments (*n* = 109; 1N [*n* = 32 × 1 litter], 1E [*n* = 29 × 1 litter], 2N (*n* = 16 × 2 litters), 2E (*n* = 16 × 2 litters), 4N (*n* = 8 × 4 litters), 4E [*n* = 8 × 4 litters]; *P *> 0.10). Across all treatments, both udder lesion scores and teat condition worsened over the course of the trial. Based on the mixing factor, sows in the single litter groups tended (*P *= 0.05) to start with lower (less severe) initial udder lesion scores on day 1 of the trial, compared to mixed litter groups (Figure 1). Furthermore, the average change in udder lesion scores *(final lesion score—initial lesion score)* tended to differ between treatments due to the mixing factor (*P *= 0.08); the udder lesion scores of single litter sows tended to worsen more throughout the trial compared to sows from the groups of two litters (*P *= 0.08) but did not differ from the groups of four litters (*P *> 0.10). In terms of teat condition, the incidence (developed during the trial) of teat infections were higher in the mixed litter groups of 2 litters mixed (*P *= 0.02) and 4 litters mixed (*P *= 0.002) when compared to the single litter groups. Additionally, the incidence of split teats was higher in sows in the 4 mixed litter groups (*P *= 0.04) compared to the single litter groups. However, the incidence of nonfunctional teats, amputated teats, and scabs was not different between treatments (*P* > 0.10; *n* = 109; 1N [*n* = 32 × 1 litter], 1E [*n* = 29 × 1 litter], 2N [*n* = 16 × 2 litters], 2E [*n* = 16 × 2 litters], 4N [*n* = 8 × 4 litters], 4E [*n* = 8 × 4 litters]).

Our results are similar to those presented by Van Kerschaer et al. (2021), who found that mixing litters of piglets had no impact on udder lesion scores which is also consistent with our findings. When sows suckle larger litters, it is expected that they would be more likely to develop teat lesions due to more teats being used by piglets during each nursing bout or due to the increased fighting between piglets in larger groups (Norring et al., 2006). This supports the perception of producers who selected large litter size as the highest risk factor attributed to sow teat lesions (Chou et al., 2022). However, since mixing litters involved removing dividers between adjacent pens, there are more piglets across more sows which may explain why the lesion scores of sows in our mixed litter groups did not worsen as much as the single litter sows. Although Camerlink et al. (2018) suggest that mixing litters preweaning can reduce sow teat condition, supported by our results of having more infected and split teats present in sows with litters that were socialized, it is also possible that not all teat lesions are due to direct piglet effects (fighting for and biting of teats). Teat lesions can be a result of ventral lying which increases in later lactation as “a way for sows to avoid stimulus from her piglets” (Norring et al., 2006) so it would be expected that larger groups of piglets disturb the sow more and therefore would result in more ventral lying. Although we did not record sow behavior or pen hygiene as part of this study, the higher incidences of specific teat conditions (inflamed and split teats) may be due to cross-sucking or reasons beyond the scope of this study and may be a concern with mixing groups on farm initially (Camerlink et al., 2018). Although mixing litters results in more piglets around the sow at a given time and may influence sow behavior, it did not negatively impact the sow’s fitness or piglet performance as a result. Contrarily, our results indicate that mixing piglets from adjacent pens may lessen the worsening of udder condition during lactation, providing a positive indication for producers who are considering mixing 2 or 4 litters before weaning in commercial settings. More research is needed to determine the cause of teat inflammation and split teats to determine how our results may influence management decisions in the future. Additionally, our results based on the presence or absence of enrichment in the preweaning environment are consistent with the results that were found in other studies (Lewis et al., 2006; Swan et al., 2021): there were no significant differences in udder or teat lesion scores between treatments with or without enrichment (*P* > 0.10). Although enrichment may provide an outlet for piglets’ natural chewing and biting behaviors and redirect some behaviors away from the sow (Lewis et al., 2006), piglets are still highly motivated to perform teat-seeking behaviors, establish a teat order, and nudge the sow’s udder to stimulate milk let down. Therefore, access to enrichment, such as burlap, may redirect some exploratory behaviors of piglets away from the sow’s udder/teats (Chou et al., 2022) but only numerical differences in sow lesion scores were found in the current study as well as by Lewis et al. (2006). Literature evaluating the effect of preweaning enrichment on piglet behavior is limited however, our findings are consistent with other published literature and support the inclusion of enrichment in the preweaning environment to benefit the piglets as well as the sows (Chou et al., 2022).

### Litter Performance

Litter performance was not impacted by allowing piglets to socialize preweaning or by adding burlap to their environment. Piglet birth weights and the average number of piglets weaned per sow were the same across treatments (*P *> 0.10; Table 4).

Our results are supported by the findings of other studies finding no effect of preweaning socialization on the number of piglets weaned per sow (D’Eath, 2005; Kutzer et al., 2009). These results indicate that mixing piglets preweaning does not have a negative impact on piglet performance. Although we could not assess weaning weight in this study, others have previously reported that preweaning socializing between piglets does not result in differences in piglet body weights at weaning (Ledergerber et al., 2015; Turpin et al., 2017; Camerlink et al., 2018; Van Kercshaver et al., 2021) nor does access to enrichment (Lewis et al., 2006; Yang et al., 2018; Swan et al., 2021).

Additionally, preweaning mortality after experimental treatments were applied and over the course of the trial (day 2 ± 1 to 22 ± 4 of life) averaged 8.9% and was not different between treatments (*P *> 0.10). Van Kerschaver et al. (2021) also found no difference in mortality from 16 d prior to weaning (when their treatments were applied) up until weaning at 21 d of age and did not look further into the causes of mortality. In our results, the proportion of mortality that was due to crushing by the sow decreased as the number of litters mixed increased (> 60% of mortality in single crates, 55% to 60% of 2-crate mortality, < 50% of 4-crate mortality; *P *> 0.10). Furthermore, the proportion of piglets crushed by the sow out of the total piglets per experimental unit was significantly different between treatments (*P* = 0.02). Specifically due to the mixing factor (*P* = 0.004), with single litters having almost 10% of piglets laid on, double litters having 6% of piglets laid on, and groups of 4 litters having 4% of piglets laid on. This may be from an increased space allowance per pig and more safe space available away from the sow after we removed the dividers separating the farrowing pens. Furthermore, in the sow barn used in this study, one heat mat was shared between two adjacent crates so if the dividers that spanned across the heat mats were removed, it would increase the available space on the heat mat, (by an average of 65 cm^2^ per litter in the groups of 2 litters mixed and 93 cm^2^ per litter in the four litter groups) providing an alternative heat source to the sow’s body heat for more piglets, thereby reducing the risk of the piglets being crushed by the sow.

These results are encouraging for the implementation of both object and social enrichment within the industry because the piglets’ preweaning performance was not negatively impacted, yet there is the potential to better prepare the piglets for the weaning transition.

## Conclusion

Our treatment factors allowed adjacent litters to socialize preweaning (as groups of 2 or 4 litters) and/or interact with burlap as object enrichment. Overall, treatment effects were neutral in terms of litter performance, piglet lesions, and many behaviors before weaning. While in the groups of enriched piglets, fewer displacements were observed around the pen and there tended to be fewer biting behaviors exhibited and less pen object manipulation, they also tended to displace piglets at the teat more frequently than non-enriched groups. Furthermore, the groups of piglets that were not mixed were observed to be the least active, use the burlap the least, and the sows were found to have the most worsening udder lesion scores when compared to the mixed litters. However, there was an increase in observed cross-sucking among larger groups of mixed litters. Additionally, we addressed many potential concerns with preweaning socialization and object enrichment, most notably, the feasibility, labor requirements, and risk to slurry manure systems. Overall, piglet and sow performance and behavior are not negatively impacted, and ultimately, their welfare may be improved preweaning.
